# Association of *KIRREL3* Intronic Polymorphisms with Carcass Traits, Meat Quality, and Muscle Fatty Acid Composition in Woking Cattle

**DOI:** 10.3390/ani16182858

**Published:** 2026-09-11

**Authors:** Xiaotong Luo, Hongliang Liu, Xuanyu Li, Chunyu Li, Congcong Zhang, Dong Li, Yongsheng Yu, Jian Wu

**Affiliations:** 1 Jilin Academy of Agricultural Sciences, Changchun 130033, China; 15568528735@163.com (X.L.); aksilirwy@163.com (H.L.); lixuanyu123456@126.com (X.L.); 18104333710@163.com (C.L.); cczhang0204@163.com (C.Z.); 2 Changchun Haoyue Co., Ltd., Changchun 130013, China; lidong8912@foxmail.com

**Keywords:** Woking cattle, *KIRREL3* gene, SNP, intronic polymorphism, meat quality traits, fatty acids, tissue expression, marker-assisted selection

## Abstract

Intramuscular fat (IMF) deposition drives premium beef value, but phenotypic selection is costly and slow. Although genome-wide studies have linked the *KIRREL3* region to meat quality, whether intronic variants directly affect carcass traits and retain predictive power across physiological stages remains unproven. We genotyped two intronic SNPs (g.29756010C>T and g.29756149C>T) in Woking cattle, a high-quality beef breed developed in Jilin Province, China (*n* = 473; 226 and 206 heifers at 36 and 48 months, respectively, plus an independent validation cohort of *n* = 41), assessed for fatty acid composition, meat quality, and multi-tissue gene expression. Both loci were polymorphic and in Hardy–Weinberg equilibrium. At 36 months, TT individuals showed higher dressing percentage, rib thickness, and meat grade than CC at both loci. By 48 months, the 63-bp locus retained broader associations with carcass traits, whereas the 203-bp locus effects weakened. In the validation group, CC animals had higher IMF and muscle *KIRREL3* expression than TT, yet received lower meat grades. Five fatty acids differed significantly among genotypes after Benjamini–Hochberg correction (q < 0.05), whereas the dominant fatty acids (C16:0, C18:1n9c, C18:0, C18:2n6c) showed no proportional differences. *KIRREL3* was most highly expressed in the kidney among eight tissues examined. These findings identify the 63-bp site as a candidate marker for meat quality in local Chinese cattle, with potential for marker-assisted selection pending confirmation in larger cohorts and functional studies.

## Simple Summary

This study examined whether two SNPs in the *KIRREL3* gene are associated with meat quality in Woking cattle, a high-quality beef breed developed in Jilin Province, China (*n* = 473, plus an independent validation cohort of *n* = 41). At the 63-bp site (g.29756010C>T), TT animals showed higher dressing percentage, rib thickness, and meat grade than CC animals at both 36 and 48 months of age, and higher intermuscular fat thickness at 36 months. In the validation group, CC animals had higher intramuscular fat content and muscle *KIRREL3* mRNA levels than TT, yet received lower meat grades. Five fatty acids differed significantly among genotypes after Benjamini–Hochberg correction (q < 0.05), including cis-10-pentadecenoic acid (C15:1), γ-linolenic acid (C18:3n6), and nervonic acid (C24:1); the dominant fatty acids (C16:0, C18:1n9c, C18:0, C18:2n6c) showed no significant differences. Together, these results identify the 63-bp site as a candidate marker for early marker-assisted selection to improve meat quality in Woking cattle, pending confirmation in larger cohorts and functional studies.

## 1. Introduction

Intramuscular fat (IMF) deposition is the single most important determinant of beef market value, governing tenderness, juiciness, and flavor [1]. IMF is polygenic and environmentally sensitive [2]. Consequently, conventional phenotypic selection requires rearing animals to slaughter age. In premium Chinese beef populations, this period often exceeds 36 months. Producers currently lack any means of early genetic screening for marbling potential. This inability to identify superior candidates before years of costly feeding represents a major economic constraint in beef improvement [3,4]. While breeds such as Angus and Wagyu have established marbling benchmarks through decades of selective breeding [5], Chinese indigenous populations including Woking cattle remain largely unexplored at the molecular level, and validated candidate-gene markers for these breeds are scarce.

Woking cattle is a local breed prized for marbled meat. Achieving market-grade marbling in this breed typically requires a finishing period of more than 36 months. However, meat quality can only be assessed post-slaughter. Consequently, producers invest years of feed and labor in animals that may ultimately grade poorly. They lack opportunities for early genetic screening. Validated candidate-gene markers for Woking cattle are scarce. Candidate-gene association analysis offers a practical first step toward enabling marker-assisted selection for marbling potential.

While lipogenic genes such as *FASN*, *SCD*, and *PLAG1* have been extensively characterized as drivers of IMF and fatty acid profiles in Hanwoo and Japanese Black cattle [6], the *KIRREL3* region offers a distinct, underexplored entry point. *KIRREL3* (kin of IRRE-like 3, *NEPH2*) encodes a homophilic cell adhesion molecule of the immunoglobulin superfamily [7,8,9], initially characterized for its role in neuronal synapse formation [10]. Human genetics has since linked *KIRREL3* mutations to autism spectrum disorder [11], and related clinical phenotypes [12], and aberrant expression has been implicated in cancer progression [13]. More pertinent to the present context, *KIRREL3* is expressed in adult human skeletal muscle, where tissue-specific alternative splicing generates multiple transcript variants [14]. In livestock, genome-wide association studies have further nominated *KIRREL3* as a candidate gene for body conformation traits in commercial pigs [15]. At the functional level, *KIRREL3* co-expresses with the alpha3 subunit of Na^+^, K^+^-ATPase [16], suggesting a role in cellular energy metabolism and signal transduction. Notably, selection signals in the BTA29 interval encompassing *KIRREL3* have been identified in beef cattle [17] and Japanese Wagyu [18], yet these genome scan findings remain untranslated into validated marker-trait associations. This combination of genomic selection signals, muscle expression, and metabolic relevance led us to prioritize *KIRREL3* over better-known lipogenic candidates for association testing in Woking cattle.

No study has tested whether *KIRREL3* marker effects persist across the finishing period: most candidate-gene studies rely on a single time point, whereas IMF accumulates continuously and metabolic pathway activity shifts markedly around 36 months of age [19]. The same marker may therefore behave differently at 36 and 48 months. In Hanwoo, an integrated approach has proven effective for refining candidate markers [20]. This approach combines polymorphism analysis, fatty acid profiling, and tissue expression. Intronic SNPs can modulate tissue-specific expression via regulatory elements, even though they remain silent at the protein level [21]. Such features make integrated approaches particularly relevant for evaluating non-coding variation. However, they have not yet been applied to *KIRREL3* in Chinese local cattle.

In the present study, we genotyped two intronic *KIRREL3* SNPs in Woking cattle by direct sequencing. We performed association tests at 36 and 48 months of age. We also incorporated an independent validation cohort, muscle fatty acid profiling, and multi-tissue expression analysis. It is important to note one limitation. Candidate-gene association analysis identifies genetic markers statistically linked to phenotypic traits. However, it does not by itself prove that the identified variants are causal mutations. It also cannot prove that these variants directly alter gene function [3,4]. Despite this limitation, our objective was clear. We sought to identify markers robust enough to support early assisted selection.

## 2. Materials and Methods

### 2.1. Animals, Phenotypic Data, and Sample Collection

All animals were sourced from Changchun Haoyue Co., Ltd. (Luyuan District, Changchun, China), a commercial beef cattle breeding and processing facility. Animals were raised under standard commercial feeding conditions and slaughtered following routine halal commercial practices (Table 1). Blood samples were collected via tail venipuncture prior to slaughter, and tissue samples were collected post-slaughter. Body weight was determined by means of a platform scale (Hi-Hog Farm & Ranch Equipment Ltd., Calgary, AB, Canada). No animals were euthanized specifically for this study. This study was approved by the Science and Technology Ethics Review Committee of Jilin Academy of Agricultural Sciences (Changchun, China). 

A total of 432 healthy Woking females were used: 226 at 36 months of age (body weight 798.71 ± 28.93 kg) and 206 at 48 months of age (818.66 ± 30.52 kg). All animals were raised under uniform feeding conditions (total mixed ration, ad libitum water, standard finishing diet meeting NRC requirements).

Before slaughter, 10 mL of blood was collected into EDTA-coated tubes (BD Vacutainer, Franklin Lakes, NJ, USA), and live weight was recorded. After slaughter, longissimus dorsi (LD) muscle, subcutaneous backfat, heart, liver, spleen, lung, kidney, and abdominal fat were collected, snap-frozen in liquid nitrogen, and stored at −80 °C until analysis.

To validate the association at the 63-bp site, 41 healthy females were selected from a cohort of 986 animals with known *KIRREL3* genotypes, stratified to achieve balanced representation across the three genotypes (CC, TC, TT). Longissimus dorsi samples were taken for fatty acid profiling, meat quality phenotyping, and *KIRREL3* mRNA quantification. Strip loin samples from the 12th–13th rib of the left carcass were transported to the laboratory in a cooler at 0–4 °C within 2 h for meat quality analysis. Carcass measurements were performed at the 12th–13th rib interface of the left half-carcass. At this location, the longissimus dorsi muscle is exposed in cross-section, permitting direct measurement of rib meat thickness (the vertical depth of the longissimus dorsi muscle), subcutaneous backfat thickness (the depth of fat covering the lateral surface of the longissimus dorsi), and intermuscular fat thickness (the depth of fat deposited between the longissimus dorsi and adjacent muscle groups). All measurements were taken on the left carcass side using a calibrated ruler, with the cut surface held perpendicular to the vertebral column.

#### Carcass Measurements and Meat Quality Assessment

Carcass grade was evaluated on the cross-section of the longissimus dorsi muscle at the 12th–13th rib interface by three trained graders according to the enterprise grading protocol of Changchun Haoyue Co., Ltd. The grading system integrated marbling score (visual assessment of intramuscular fat distribution density and fineness), meat color, fat color, and physiological maturity into a composite score ranging from 1.0 to 5.0, where higher scores indicate superior quality. Notably, the final grade was determined primarily by marbling pattern (the fineness and evenness of intramuscular fat distribution) rather than by total IMF content alone. Assessors were blinded to genotype information. Meat quality traits were determined on the longissimus dorsi muscle after 24 h of aging at 4 °C: Intramuscular fat (IMF) content was determined by Soxhlet extraction following GB 5009.6-2016 [22] and expressed as a percentage of fresh muscle weight. Moisture content was determined by the direct drying method (GB5009.3-2016 [23]). Crude protein was determined by the Kjeldahl method (GB5009.5-2016 [24]). Meat color (L*, a*, and b* values) was measured using a chroma meter (Konica Minolta CR-400; Konica Minolta, Osaka, Japan) with a D65 light source, 10° observer angle, and an 8 mm aperture, calibrated against a standard white tile. Three random readings were taken on the freshly cut muscle surface and averaged. pH was measured at 24 h post-mortem using a portable pH meter (Matthäus GmbH & Co. KG, Eckelsheim, Germany) inserted into the geometric center of the muscle. Drip loss was measured as the percentage weight loss of an 80 g muscle sample suspended in a sealed plastic bag at 4 °C for 24 h. Pressurized water loss (pressing loss) was determined by the filter-paper press method using a Runhu RH-1000 meat press (Guangzhou Runhu Instrument Co., Ltd., Guangzhou, China) at 35.0 kg for 5 min, expressed as the percentage of weight loss relative to the initial sample weight. Cooking loss was measured after heating vacuum-sealed samples in a water bath (Model DK-8D, Shanghai Yiheng, Shanghai, China) at 80 °C until the core temperature reached 70 °C, followed by cooling to room temperature and calculating the percentage weight loss. Cooked meat rate was calculated as the ratio of cooked weight to raw weight. Centrifugal loss was determined by centrifuging a standardized meat sample at 9000 rpm for 10 min at 4 °C in a Dynamica V14R centrifuge (Dynamica Scientific Ltd., Livingston, UK), expressed as percentage weight loss. Tenderness was evaluated as Warner-Bratzler shear force (WBSF). Cooked samples were cooled to room temperature, and cylindrical cores (1.27 cm diameter) were extracted parallel to the muscle fiber orientation and sheared perpendicular to the fibers using a Lloyd TA1 texture analyzer (Lloyd Instruments Ltd., Bognor Regis, UK) equipped with a Warner-Bratzler blade at a crosshead speed of 200 mm/min. Peak shear force was recorded in Newtons (N).

### 2.2. Genomic DNA and Total RNA Extraction

Genomic DNA was extracted from whole blood using the UE Blood Genomic DNA Miniprep Kit (UElandy, Suzhou, China, Lot. No. 251029KC3) according to the manufacturer’s protocol. DNA concentration and purity (A260/A280) were measured on a NanoDrop ND-1000 spectrophotometer (Thermo Fisher Scientific, Waltham, MA, USA), and DNA integrity was verified by 1.0% agarose gel electrophoresis. Only samples with A260/A280 ratios of 1.8–2.0 and intact high-molecular-weight bands were retained. DNA was stored at −20 °C.

Total RNA was isolated from kidney, backfat, liver, longissimus dorsi, abdominal fat, heart, spleen, and lung. using TRIzol reagent (Ambion, Austin, TX, USA, Lot. No. 350103) following the manufacturer’s instructions. RNA concentration and purity (A260/A280) were assessed using a Quawell-Q5000 UV spectrophotometer (Quawell Technology, San Jose, CA, USA), and integrity was verified by 2.0% agarose gel electrophoresis (2 μL loaded). Samples with A260/A280 ratios of 1.8–2.0 and distinct 28S/18S rRNA bands were retained for downstream analysis. RNA was stored at −80 °C.

### 2.3. Primer Design and Specificity Validation

The bovine reference genome (ARS-UCD2.0, GCA_002263795.2) maps KIRREL3 (ENSBTAG00000050123; transcript ENSBTAT00000066392.2) to chromosome 29. The two targeted SNPs are located in the first intron at ARS-UCD2.0 coordinates 29:g.29756010C>T and 29:g.29756149C>T. A single primer pair was designed with Primer Premier 5.0 (Premier Biosoft, Palo Alto, CA, USA) to amplify a 603 bp fragment spanning the 63-bp (g.29756010C>T) and 203-bp (g.29756149C>T) sites. Primer specificity was confirmed by in silico PCR against the ARS-UCD2.0 reference genome and by the presence of a single band of expected size on 1.5% agarose gel.

For RT-qPCR, primers were designed against the *KIRREL3* coding sequence (RefSeq XM_025286398.3), with β-actin (NM_173979) as the internal reference. Primer pairs were validated by standard PCR to ensure a single amplicon of expected size, and melting curve analysis (single peak) confirmed the absence of primer dimers. All primers were synthesized by Genewiz (Suzhou, China). Sequences are listed in Table 2.

### 2.4. PCR Amplification and Sanger Sequencing

PCR was performed in a 20 μL reaction containing 10 μL of 2× Es Taq Master Mix (Dye) (CWBiotech, Beijing, China), 0.5 μL each primer (10 μmol/L), 1 μL DNA template (50 ng/μL), and 8 μL RNase-free water. The cycling conditions were: initial denaturation at 94 °C for 4 min; 35 cycles of 94 °C for 30 s, 56 °C for 40 s, and 72 °C for 30 s; and a final extension at 72 °C for 7 min. Products were checked on a 1.5% agarose gel. Clean amplicons were purified and submitted to Genewiz (Suzhou, China) for bidirectional Sanger sequencing.

Genotypes were called from chromatograms using SnapGene software (v6.0.2; Insightful Science, San Diego, CA, USA) by two independent operators. Heterozygous peaks were identified by the presence of overlapping nucleotide signals at the SNP position. Ambiguous calls were re-amplified and re-sequenced. Ambiguous calls that failed re-amplification and re-sequencing were excluded from analysis. The overall call rate exceeded 98% across both SNPs.

### 2.5. Fatty Acid Analysis

Muscle samples were analyzed at the Risk Assessment Laboratory for Agricultural Product Quality and Safety, Ministry of Agriculture and Rural Affairs (Changchun, China). Freeze-dried and ground muscle (0.03 g) was subjected to lipid extraction by acid hydrolysis according to GB 5009.168-2016 [25], using petroleum ether-anhydrous diethyl ether (1:1, *v*/*v*). After evaporation at room temperature, methyl esterification was performed following the transesterification protocol described in the same standard. The residue was dissolved in 4 mL n-hexane. Then 200 μL of 2 mol/L KOH–methanol was added. The mixture was shaken vigorously for 30 s. After neutralization with sodium bisulfate, the supernatant was collected for GC-MS/MS analysis.

Fatty acid methyl esters (FAMEs) were analyzed on an Agilent 8890 GC coupled to a 7000D triple quadrupole MS/MS (Agilent Technologies, Santa Clara, CA, USA), fitted with a DB-FastFame capillary column (30 m × 250 μm × 0.25 μm) (Agilent Technologies, Santa Clara, CA, USA). The injector was held at 280 °C in splitless mode with a helium carrier flow of 1.0 mL/min. The oven temperature program was: 80 °C for 0.5 min; ramp to 165 °C at 40 °C/min, hold 1 min; ramp to 230 °C at 4 °C/min, hold 4 min; post-run at 260 °C for 5 min. The electron ionization (EI) source was set to 70 eV with a 2 min solvent delay. A 37-component FAME standard (Merck, Darmstadt, Germany) was used for external calibration and peak identification. Results are expressed as relative weight percentage (% of total identified fatty acids) based on fresh tissue weight. The sum of all identified fatty acids in each sample was normalized to 100% for presentation; statistical analyses were performed on the raw concentration data (g/100 g).

### 2.6. Reverse Transcription and RT-qPCR

Prior to reverse transcription, total RNA was treated with DNase I (RNase-free, Sangon Biotech, Shanghai, China) to eliminate genomic DNA contamination. First-strand cDNA was synthesized from 1 μg of total RNA in a 20 μL reaction using the PrimeScript RT Master Mix (Sangon Biotech, Shanghai, China; containing oligo(dT)18 and random hexamer primers) according to the manufacturer’s protocol: 37 °C for 15 min, followed by 85 °C for 5 s. cDNA was diluted 1:5 and stored at −20 °C.

Real-time PCR was performed on a LightCycler 96 (Roche, Basel, Switzerland) using SYBR Green I chemistry. Each 20 μL reaction contained 10 μL of 2× SYBR Green Master Mix, 0.4 μL each of 10 μmol/L forward and reverse primers, 1 μL of diluted cDNA template, and RNase-free water. The thermal profile was: pre-denaturation at 95 °C for 3 min; 40 cycles of 95 °C for 10 s and 60 °C for 30 s. A melting curve analysis (65–95 °C, 0.1 °C/s) was performed at the end of each run to confirm the specificity of amplification (single peak). Each sample was run in triplicate technical replicates, and the mean Ct value was used for quantification. β-actin served as the reference gene, and relative KIRREL3 expression was calculated by the 2^−ΔΔCt^ method [26], with heart tissue as the calibrator tissue.

### 2.7. Statistical Analysis

Genotype frequencies, allele frequencies (p), expected heterozygosity (He), polymorphism information content (PIC), and effective allele number (Ne) were calculated according to Hartl [27]. Hardy–Weinberg equilibrium (HWE) was tested using Pearson’s chi-squared test; when any expected genotype count was less than 5, McDonald’s exact test was applied [28].

Associations between genotype and growth, carcass, and meat quality traits were assessed by one-way ANOVA in Python (v3.12; Python Software Foundation, Wilmington, DE, USA) using the SciPy library (v1.11.4) with genotype as the fixed factor. For each trait, three pre-specified genetic contrasts were evaluated within each age group using Tukey’s honestly significant difference (HSD) post-hoc test: (i) CC versus TT to estimate the homozygous allelic effect; (ii) CC versus CT to assess the heterozygous effect relative to the wild-type; and (iii) CT versus TT to test for dominance deviation. Data are presented as mean ± SD, and group differences are marked with letters (a, b, ab) based on Tukey’s HSD pairwise comparison results. *p* < 0.05 was considered statistically significant.

For RT-qPCR, relative expression differences among tissues and among genotypes were analyzed by one-way ANOVA. The analysis was performed in Python (v3.12; Python Software Foundation, Wilmington, DE, USA) using the SciPy library (v1.11.4),. Tukey’s honestly significant difference (HSD) post-hoc test was used for pairwise comparisons. Normality and homogeneity of variance were verified by the Shapiro–Wilk test and Levene’s test, respectively. *p* < 0.05 was considered statistically significant.

For carcass and meat quality traits, data were first tested for normality (Shapiro–Wilk test) and homogeneity of variance (Levene’s test). All traits met the assumptions for parametric analysis; therefore, one-way ANOVA followed by Tukey’s HSD post-hoc test was applied. For fatty acid composition, many individual fatty acids exhibited non-normal distributions and heterogeneous variances; consequently, the Kruskal–Wallis H test followed by Dunn’s test with Benjamini–Hochberg correction was applied using the scikit-posthocs package. The choice of test was determined by the distributional properties of each dataset, not by the research question. *p* < 0.05 was considered significant for parametric tests, and q < 0.05 for BH-corrected non-parametric comparisons. Data are presented as mean ± SD, with group differences marked by letters.

### 2.8. In Silico Virtual Knockout and Functional Impact Prediction

Because the GWAS signal for *KIRREL3* showed an unexpected negative correlation with BMS, we used an in silico approach to predict what happens when this gene is knocked out. The prediction combined three sources: first, population-genomic selection signals from Wagyu cattle GWAS (BTA29: g.29756010C>T, *p* < 0.05) showing a negative correlation between KIRREL3 expression and meat marbling score (BMS); second, functional annotation from the Ig-superfamily adhesion literature and myogenesis studies indicating that KIRREL3 mediates homophilic trans-cellular adhesion, regulates myoblast morphology, and is required for MyoD-dependent myotube formation; and third, network perturbation prediction based on established myogenic (*MYOD, MYOG, MEF2C*) and adipogenic (*PPARG, FABP4, FASN, SCD*) regulatory cascades in bovine skeletal muscle. Candidate differentially regulated genes (DRGs) after virtual KO were predicted according to their established regulatory relationships with KIRREL3-mediated cell adhesion and muscle-fat infiltration dynamics. The predicted impact on beef marbling was further interpreted under the “adhesion barrier hypothesis,” which posits that KIRREL3 expression on myoblast membranes acts as a physical barrier limiting preadipocyte infiltration into muscle bundles.

### 2.9. Data Availability Statement

The raw sequencing chromatograms and phenotypic datasets generated during this study are available from the corresponding author upon reasonable request. Accession numbers for deposited sequence data will be provided during the review process.

## 3. Results

### 3.1. Sequencing and PCR Amplification Results

**Figure 1:**
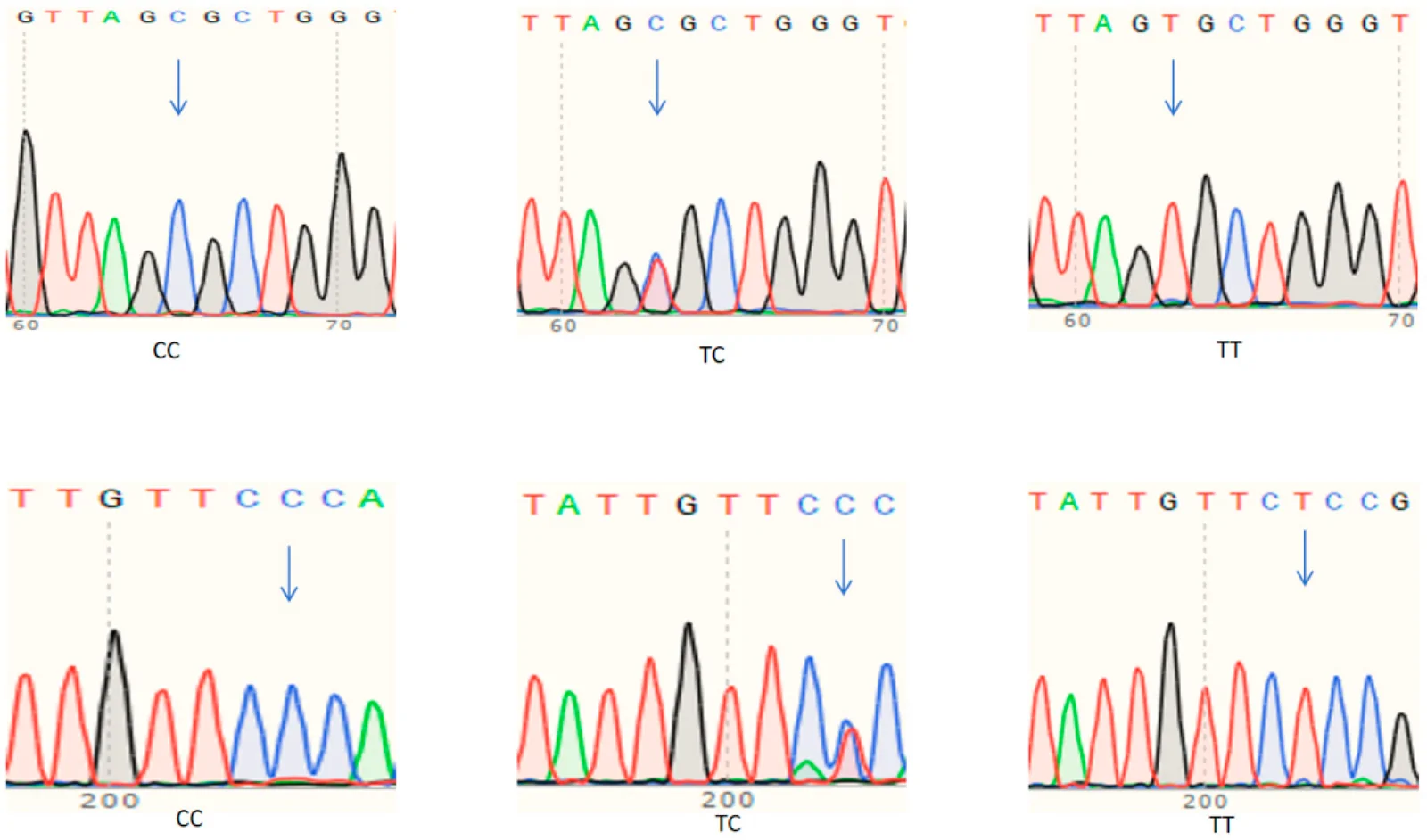
Sanger sequencing chromatograms of *KIRREL3* intronic SNPs. **Upper panel**: g.29756010C>T (63-bp site); **Lower panel**: g.29756149C>T (203 bp site).

**Figure 2:**
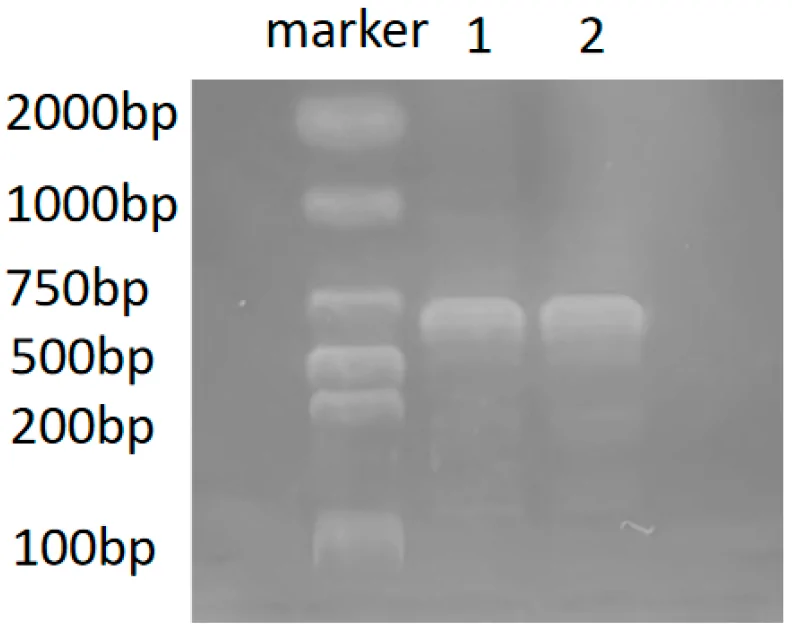
Agarose gel electrophoresis of *KIRREL3* PCR amplification products in Woking cattle.

Direct sequencing of the 603 bp KIRREL3 intronic fragment revealed two SNPs (Figure 1): g.29756010C>T (63-bp site) and g.29756149C>T (203-bp site). At the 63-bp site, CC showed a single C peak, TC showed overlapping C/T peaks, and TT showed a single T peak (Figure 1, upper panel). The 203-bp site displayed the same chromatogram pattern (Figure 1, lower panel). PCR amplification produced a single clean band at approximately 603 bp, consistent with the expected size (Figure 2).

### 3.2. Polymorphism Analysis

Both SNPs were moderately polymorphic (0.25 < PIC < 0.50). At the 63-bp site, the CT heterozygote was most frequent (51.77%), followed by CC (29.20%) and TT (19.03%). Allele frequencies were 0.5509 (C) and 0.4491 (T); observed heterozygosity (Ho), expected heterozygosity (He), effective allele number (Ne), and PIC were 0.52, 0.49, 1.98, and 0.37, respectively. At the 203-bp site, TC again predominated (52.94%), followed by CC (31.86%) and TT (15.20%). Allele frequencies were 0.5833 (C) and 0.4167 (T); Ho, He, Ne, and PIC were 0.53, 0.49, 1.95, and 0.37, respectively. Neither site deviated from Hardy–Weinberg equilibrium (63-bp: χ^2^ = 0.48, *p* = 0.487; 203-bp: χ^2^ = 1.62, *p* = 0.203), indicating a stable genetic structure without evidence of strong selection, inbreeding, or drift (Table 3).

### 3.3. Association Analysis of Polymorphisms with Carcass Traits

#### 3.3.1. 36-Month Group

At the 63-bp site, genotype significantly affected dressing percentage, rib thickness, intermuscular fat thickness, and meat grade (all *p* < 0.05, one-way ANOVA). Tukey’s HSD post-hoc tests revealed that TT individuals exceeded CC in dressing percentage, intermuscular fat thickness, and meat grade. For rib thickness, TT was significantly thicker than CC, while TC was intermediate and did not differ significantly from either CC or TT (Table 4).

At the 203-bp site, TT again exceeded CC in dressing percentage (*p* < 0.05), rib thickness (*p* < 0.05), and meat grade (*p* < 0.05). The pattern for rib thickness mirrored that at the 63-bp site: TT was significantly thicker than CC, while CT was intermediate and did not differ significantly from either group. For meat grade, TT was significantly higher than both CC and TC. Notably, intermuscular fat thickness showed no significant association at this site (*p* > 0.05) (Table 4).

#### 3.3.2. 48-Month Group

By 48 months, the 63-bp site showed significant genotype effects on dressing percentage, meat grade, rib thickness, and subcutaneous fat thickness (all *p* < 0.05, one-way ANOVA). Post-hoc analysis indicated that TC and TT exceeded CC in meat grade and rib thickness (both *p* < 0.01), while TT remained higher than CC in dressing percentage (*p* < 0.05). Notably, subcutaneous fat thickness was highest in TC, which significantly exceeded TT (*p* < 0.05), whereas CC was intermediate and did not differ significantly from either TC or TT, suggesting a heterotic effect.

At the 203-bp site, TT maintained a higher dressing percentage (61.68 ± 1.77%) than CC (60.64 ± 1.65%, *p* = 0.028), with TC intermediate (61.32 ± 2.20%) and not differing from either. The same pattern held for rib thickness: TT (7.81 ± 0.66 cm) > CC (7.38 ± 0.68 cm, *p* = 0.021), TC intermediate (7.65 ± 0.77 cm) (Table 5). For grade, TT exceeded both TC and CC (*p* < 0.05), whereas TC and CC were not significantly different from each other. The 63-bp site broadened its influence at 48 months, with TC and TT both outperforming CC. In contrast, the 203-bp site retained only limited associations, with significant effects for dressing percentage and rib thickness, and a weaker association with grade (where only TT differed from TC).

### 3.4. Fatty Acid Composition Among Genotypes

Thirty-three fatty acids were detected at or above the limit of detection (LOD) across the three genotype groups (Table 6). Of these, 32 were quantifiable in all three groups, and tridecanoic acid (C13:0) was detected in the TC and TT groups but not in the CC group (ND). Four acids (C4:0, C6:0, C8:0, and C11:0) were below the LOD in all samples and were excluded from further analyses. The overall fatty acid profiles were dominated by oleic acid (C18:1n9c, ~22–25%), palmitic acid (C16:0, ~17–18%), stearic acid (C18:0, ~15–16%), and linoleic acid (C18:2n6c, ~10–11%). We applied Benjamini–Hochberg (BH) correction for multiple comparisons. After correction, five fatty acids differed significantly among genotypes (q < 0.05; Table 6): cis-10-pentadecenoic acid (C15:1, q = 0.0194), γ-linolenic acid (C18:3n6, q = 0.0282), cis-11,14,17-eicosatrienoic acid (C20:3n3, q = 0.0008), erucic acid (C22:1, q = 0.0067), and nervonic acid (C24:1, q = 0.0000). All significant differences involved low-abundance fatty acids (<1% of total identified FAs). The dominant fatty acids (C16:0, C18:0, C18:1n9c, C18:2n6c) showed no significant differences among genotypes (all q > 0.05). The remaining twenty-two fatty acids showed no significant differences among genotypes (q > 0.05).

### 3.5. Meat Quality Traits Among Genotypes

Forty-one females from a cohort of 986 animals were randomly selected and stratified by *KIRREL3* genotype (CC, *n* = 12; TC, *n* = 20; TT, *n* = 9) for meat quality analysis (Table 7). IMF content differed significantly among genotypes (*p* = 0.0267, one-way ANOVA). Tukey’s HSD post-hoc tests showed that CC exceeded TT (*p* < 0.05), while TC was intermediate and did not differ significantly from either CC or TT. CC had significantly higher L* and b* values than TT (*p* < 0.001 and *p* < 0.05, respectively). For water-holding capacity, centrifugal loss was higher in TT (*p* < 0.05), whereas cooking loss was lower in CC (*p* < 0.01 vs. TC). TT showed significantly higher crude protein than CC (*p* < 0.01); TC was intermediate and not significantly different from either group. Pressing loss, drip loss, tenderness, and a* value showed no significant differences (*p* > 0.05), although TT had numerically lower values (Table 7).

### 3.6. Polymorphism in Prairie Red and Yanbian Cattle

To clarify the genetic background of the *KIRREL3* marker in Northeast Chinese cattle, the two SNPs were genotyped in Prairie Red (*n* = 50) and Yanbian (*n* = 50) cattle. Both breeds exhibited intermediate polymorphism (PIC = 0.37), with effective allele numbers close to 2. Observed and expected heterozygosities were similar, and Hardy–Weinberg equilibrium was maintained (*p* > 0.05), indicating a stable population genetic structure. No significant differences were found between the two breeds in genotype distribution (χ^2^ = 2.496, *p* = 0.287) or allele frequencies (χ^2^ = 2.010, *p* = 0.156). The genetic differentiation coefficient Fst was 0.0122, indicating weak differentiation. The T allele frequency was slightly higher in Prairie Red (0.59) than in Yanbian (0.48), but this difference was not statistically significant (Table 8).

### 3.7. Tissue Expression of KIRREL3

**Figure 3:**
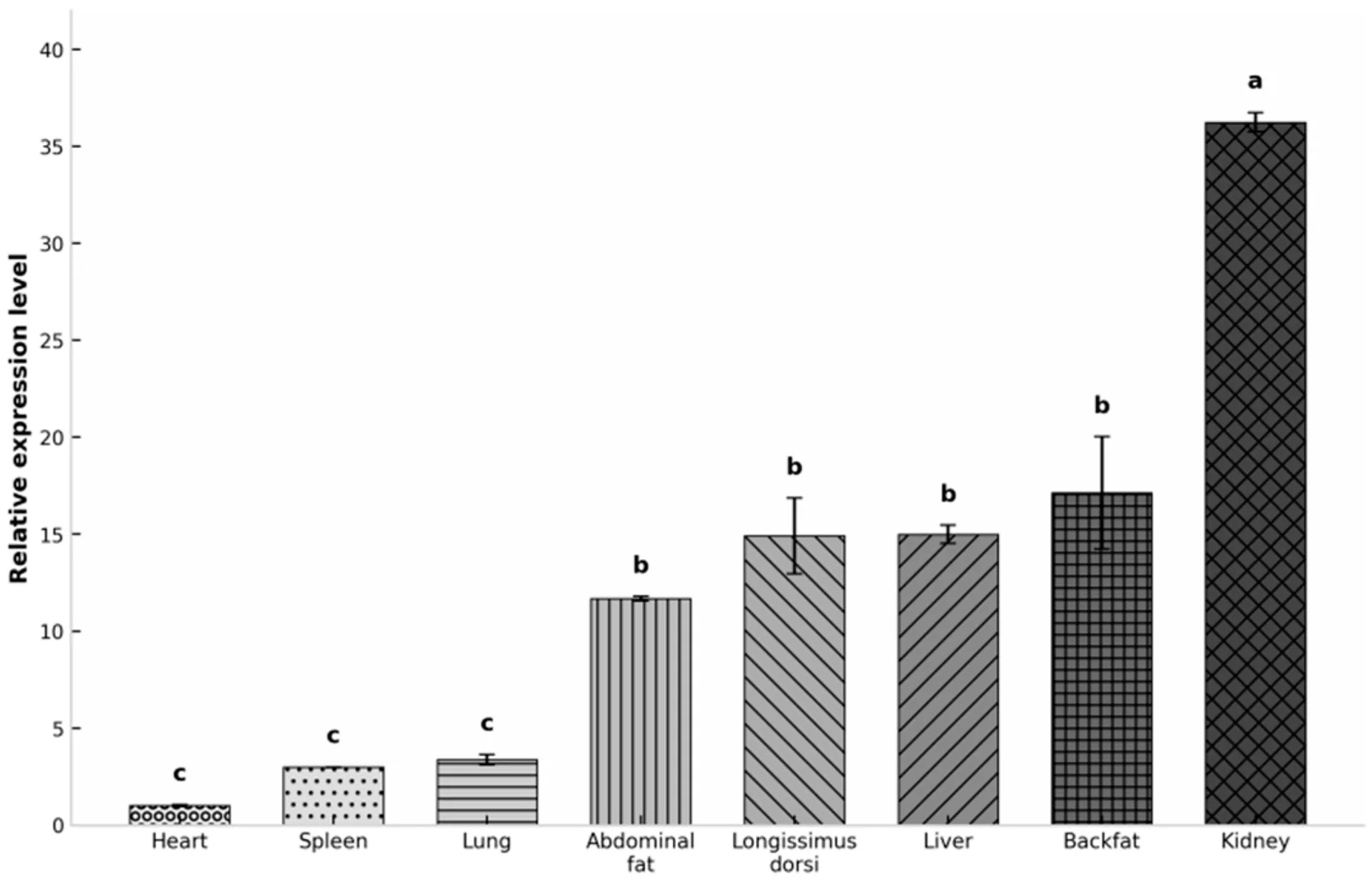
Tissue-specific expression of *KIRREL3* in Woking cattle. Relative mRNA levels were determined by RT-qPCR and normalized to β-actin, with heart tissue as the calibrator (set to 1.00). Data are presented as mean ± SD (*n* = 3). Different letters above the bars indicate significant differences among tissues (*p* < 0.05, Tukey’s HSD test): a, kidney; b, backfat, liver, longissimus dorsi, and abdominal fat; c, heart, spleen, and lung.

Quantitative PCR detected *KIRREL3* mRNA in all eight tissues examined, with heart as the calibrator (set to 1.00). Expression was markedly tissue-specific. Kidney exhibited the highest expression level (36.25-fold relative to heart, group a), followed by backfat, liver, longissimus dorsi, and abdominal fat (11.67- to 17.13-fold, group b), all of which were significantly higher than heart (*p* < 0.05, Tukey’s HSD test). Spleen and lung displayed expression levels of 2.98-fold and 3.37-fold, respectively. These tissues were grouped with heart in the lowest expression tier (group c). No statistically significant differences were detected among these three tissues (Figure 3). The small sample size (*n* = 3 per tissue) may have limited statistical power to detect modest expression differences among low-expression tissues.

### 3.8. Genotype-Specific Expression of KIRREL3 in Muscle

**Figure 4:**
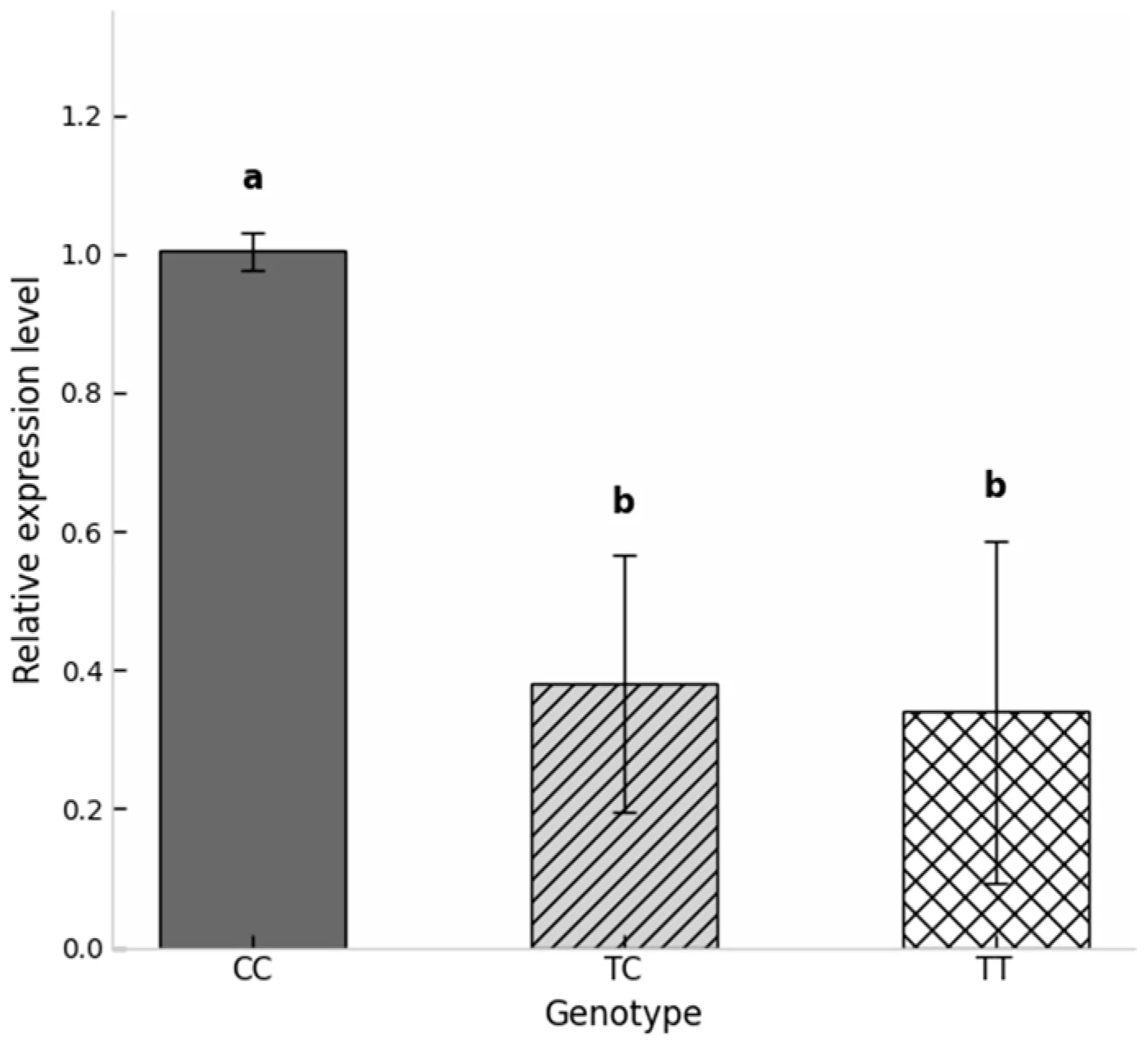
Genotype-specific expression of *KIRREL3* in the longissimus dorsi muscle of Woking cattle. Relative mRNA levels were determined by RT-qPCR and normalized to β-actin. Data are presented as mean ± SD (CC, *n* = 12; TC, *n* = 20; TT, *n* = 9). Different letters above the bars indicate significant differences among genotypes (*p* < 0.05, one-way ANOVA followed by Tukey’s HSD test): a, CC; b, TC and TT.

In muscle samples from the validation population, *KIRREL3* relative expression was significantly higher in the CC genotype than in TC and TT (*p* < 0.05), with no difference between TC and TT (*p* > 0.05) (Figure 4). Notably, CC showed the highest muscle expression but corresponded to the lowest meat grade. In contrast, TT showed the lowest expression but achieved the highest grade. This inverse pattern suggests a complex relationship between *KIRREL3* expression and marbling score, which may be antagonistic.

### 3.9. Virtual Knockout Prediction Reveals an Adhesion Barrier Mechanism for KIRREL3 in Woking Marbling

**Figure 5:**
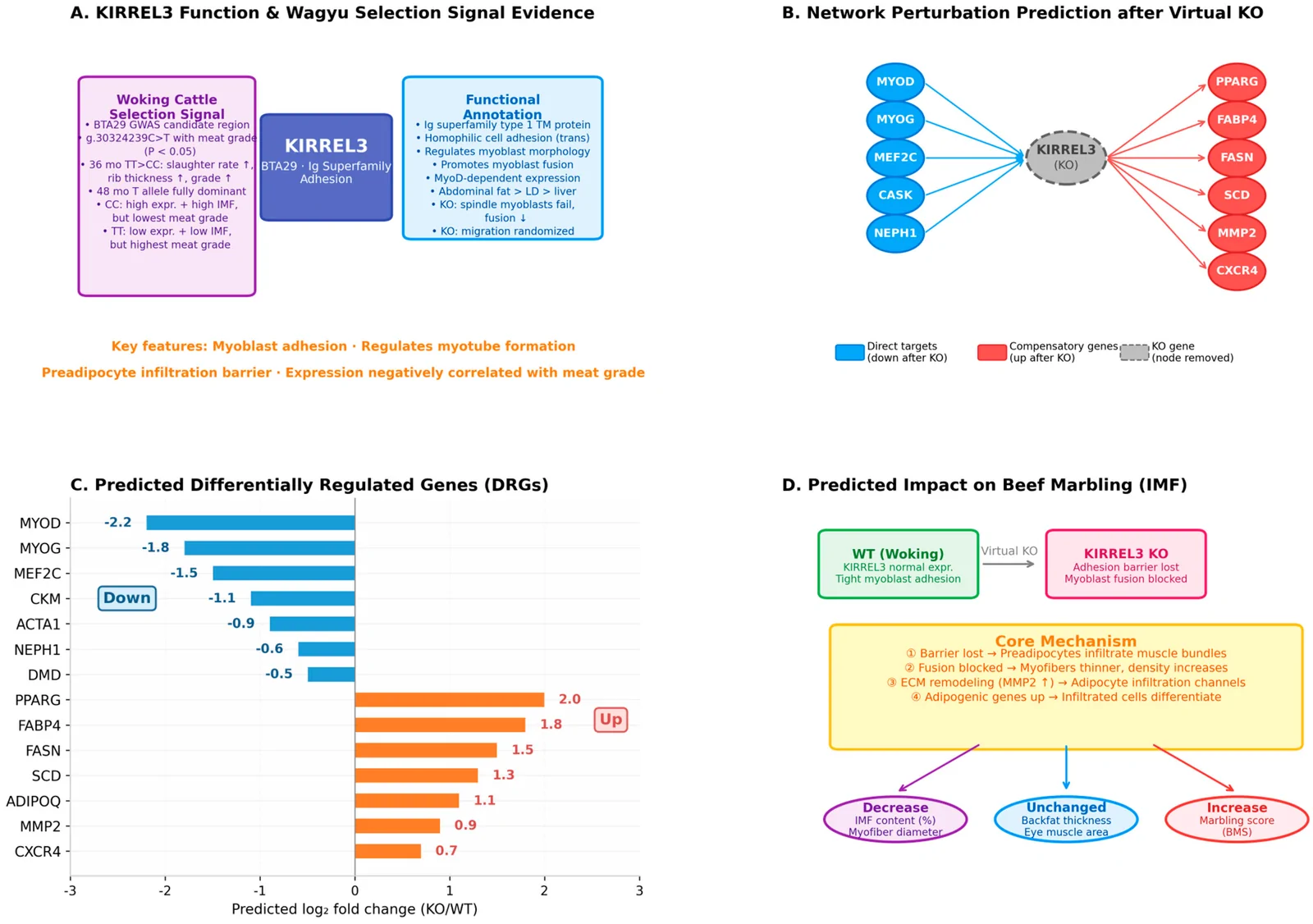
In silico virtual knockout prediction of *KIRREL3* and its impact on beef marbling in Woking. (**A**) Functional evidence and selection signals supporting KIRREL3 as a candidate gene. Left: Woking cattle GWAS signals showing the intronic SNP g.29756010C>T is significantly associated with meat grade (*p* < 0.05), with the T allele being fully dominant at 48 months. Notably, the CC genotype exhibits high *KIRREL3* expression and high intramuscular fat (IMF) content but the lowest meat grade, whereas the TT genotype shows the opposite pattern. Right: Functional annotations from literature integration indicating *KIRREL3* is an Ig-superfamily type I transmembrane protein that regulates myoblast morphology, promotes fusion, and is essential for MyoD-dependent myotube formation. (**B**) Predicted network perturbation after virtual KO. Direct target genes (blue), including the core myogenic transcription factors (*MYOD*, *MYOG*, *MEF2C*) and adhesion complex partners (*CASK, NEPH1*), are predicted to be downregulated. Compensatory genes (red) involved in adipogenesis (*PPARG*, *FABP4*, *FASN*, *SCD*), extracellular matrix remodeling (*MMP2*), and cell migration (*CXCR4*) are predicted to be upregulated. (**C**) Predicted differentially regulated genes (DRGs) displayed as log_2_ fold change (KO/WT). (**D**) Predicted impact on beef marbling (IMF). The core mechanism involves loss of the adhesion barrier, blocked myoblast fusion, enhanced ECM remodeling, and upregulation of adipogenic genes, collectively promoting preadipocyte infiltration into muscle bundles. Predicted outcomes: decreased IMF chemical content (%) and myofiber diameter; unchanged backfat thickness and eye muscle area; increased visible marbling score (BMS).

Unlike conventional IMF deposition genes, *KIRREL3* exhibited a counterintuitive expression-phenotype relationship in the Woking cattle population: individuals carrying the CC genotype at g.29756010C>T displayed the highest KIRREL3 expression and highest IMF content, yet received the lowest meat grade; conversely, TT individuals with low expression and low IMF achieved the highest grade (Figure 5A). To resolve this paradox, we performed an in silico virtual KO of KIRREL3. The network perturbation prediction indicated downregulation of core myogenic factors (*MYOD*, *MYOG*, *MEF2C*) and upregulation of adipogenic (*PPARG*, *FABP4*, *FASN*, *SCD*) and ECM-remodeling (*MMP2*) genes (Figure 5B,C). Under the adhesion barrier hypothesis, KIRREL3 loss would relax myoblast-myoblast adhesion, allowing preadipocytes to infiltrate inter-myofiber spaces while simultaneously blocking myoblast fusion, resulting in thinner myofibers and higher muscle density. Consequently, although total IMF content is predicted to decrease, the spatial redistribution of adipocytes into visible inter-fascicular regions is predicted to increase the objective marbling score (BMS) (Figure 5D).

## 4. Discussion

### 4.1. Genotype Frequencies, Allele Frequencies, and Association with Carcass Traits

The two intronic SNPs in *KIRREL3* associate with carcass quality in Woking cattle, refining the BTA29 selection signal previously detected by whole-genome resequencing [16,17]. Both loci showed intermediate polymorphism (PIC ≈ 0.37) and Hardy–Weinberg equilibrium, indicating a stable population structure without strong recent selection [4,29]. This matters because moderately polymorphic loci retain sufficient genetic variation for rapid allele-frequency shifts under directional selection [4].

At 36 months, the TT genotype outperformed CC in dressing percentage, rib thickness, and meat grade at both SNPs. By 48 months, however, the marker effects diverged: the 63 bp site (g.29756010C>T) maintained robust associations, whereas the 203 bp site (g.29756149C>T) lost its association with grade and intermuscular fat thickness. This divergence likely reflects age-related changes in metabolic pathway activity. It may also reflect linkage disequilibrium decay across the extended finishing period. These explanations are more plausible than a discrete developmental transition. Alternatively, cumulative environmental variation, management practices, or genotype-by-environment interactions across the two finishing cohorts could contribute to this shift; the term “cross-stage effect” should therefore be interpreted cautiously. The sustained effect of the 63 bp site suggests it lies closer to a core regulatory domain or exerts direct cis-regulatory activity.

Both variants are intronic. Their phenotypic effects could reflect direct regulation of *KIRREL3* expression. Alternatively, they could reflect linkage disequilibrium with neighboring functional loci within the broader BTA29 haplotype block [17]. Without regional LD profiling or conditional association analysis, *KIRREL3* should be treated as a candidate marker within a region of interest, not yet as a confirmed causal gene. Traenkner et al. [30] showed that *KIRREL3* alternative splicing is linked to its synapse-specific function. They also showed that intronic variants can alter exon-skipping efficiency. Chen et al. [31] confirmed that an intronic SNP can affect muscle development. It does so by altering transcription-factor binding. These precedents support the plausibility of intronic regulatory effects, but they do not prove causality in this system.

### 4.2. Age-Dependent Effects and Breeding Implications

By 48 months, the T allele shows complete dominance at the 63 bp site. This pattern is directly advantageous for breeding. A single T allele delivers the full phenotypic benefit. Therefore, there is no pressure to fix the allele rapidly. Breeders can preserve heterozygosity and avoid inbreeding depression. This parallels the *PLAG1* 19 bp deletion in Hanwoo, where heterozygotes already show significant improvement in body size and carcass weight [29]. Notably, neither *KIRREL3* locus affected live weight, carcass weight, or ribeye area. This suggests that selection for the T allele can improve meat quality. It can do so without penalizing growth performance [2]. This marker combines complete dominance for meat quality with neutrality for growth traits. If validated in larger cohorts and functional studies, it would be a promising candidate for marker-assisted selection programs, potentially allowing breeders to use it without risking negative correlated responses in body weight or carcass yield.

### 4.3. The Dissociation Between IMF, Fatty Acids, and Meat Grade

Muscle fatty acid composition matters for beef flavor, tenderness, and nutritional value [1,32]. High SFA levels generally hurt palatability and consumer health perception, whereas MUFA and PUFA fractions support better eating quality [1,32]. Lipogenic genes—*SCD*, *FASN*, *FABP4*, and *SREBP1* among them—are established drivers of these profiles in cattle [2,29,33,34,35,36,37].

Here we report that *KIRREL3* g.29756010C>T is significantly associated with IMF content and carcass grade in Woking cattle. The C allele was associated with higher IMF deposition. Five fatty acids differed significantly among genotypes after Benjamini–Hochberg correction (q < 0.05; Table 6): cis-10-pentadecenoic acid (C15:1), γ-linolenic acid (C18:3n6), cis-11,14,17-eicosatrienoic acid (C20:3n3), erucic acid (C22:1), and nervonic acid (C24:1). All are present at low absolute concentrations (< 1% of total identified FAs). The dominant fatty acids—oleic acid (C18:1n9c, ~22–25%), palmitic acid (C16:0, ~17–18%), stearic acid (C18:0, ~15–16%), and linoleic acid (C18:2n6c, ~10–11%)—showed no significant proportional differences among genotypes (all q > 0.05). This pattern indicates that the C allele influences total lipid accretion and meat grade without altering the overall fatty acid profile.

This dissociation between genotype and fatty acid composition contrasts with the more striking lipid phenotype: CC animals accumulated more IMF and exhibited higher muscle *KIRREL3* expression than TT, yet received lower meat grades. The paradox disappears once marbling is treated as a spatial process rather than simple bulk lipid storage. Premium marbling requires preadipocyte infiltration into the perimysium and endomysium, followed by discrete lipid droplet deposition between muscle fibers [38,39]. We propose that elevated *KIRREL3* in CC animals tightens adhesion barriers on fiber surfaces, restricting preadipocyte migration into the interstitial spaces where visible marbling forms. Lipids then accumulate in intracellular or peripheral depots that add IMF weight but not the fine, evenly distributed pattern graders value. Lower *KIRREL3* in TT may loosen those barriers, permitting the infiltrative architecture that yields higher grades at lower IMF.

Nutritionally, the genotype effect should be interpreted cautiously. Ueda et al. [5] have argued that lowering C16:0 and C18:0 while raising C18:1 and C18:3n3 improves beef’s nutritional score. In our cohort, none of these major nutritionally relevant fatty acids differed significantly; the absolute differences for the significant minor fatty acids were small (e.g., C15:1 differed by ~0.1 percentage points between CC and TC), and the overall proportional composition remained within the normal range for beef longissimus dorsi. Breeders can therefore use this marker to improve visual marbling quality without substantially altering the nutritional fatty acid signature; however, the modest elevation of specific minor FAs in CC animals warrants monitoring in selection programs.

### 4.4. Cross-Breed Validation and Marker Transferability

Both Prairie Red and Yanbian cattle showed intermediate polymorphism at the *KIRREL3* locus. The FST was only 0.0122. This indicates high genetic homogeneity among Northeast Chinese local breeds. Such homogeneity provides preliminary evidence. The 63 bp marker may be broadly applicable across these populations [40,41,42]. Chinese local cattle maintain higher genetic diversity than European commercial breeds [43]. This diversity offers a reservoir of breed-specific variation for marker-assisted selection. Baek et al. [40] identified breed-specific selective sweeps in Yanbian cattle. These sweeps are associated with environmental adaptation. They highlight the genomic diversity of this local breed. Yu et al. [41] and Wang et al. [42] reported genetic diversity patterns related to IMF in Qinchuan cattle. The *KIRREL3* locus shows similar polymorphism distributions across the three breeds. This provides preliminary evidence for marker transferability in Chinese local cattle populations.

### 4.5. Implications of the KIRREL3 Adhesion-Barrier Model for Woking Breeding

The virtual KO prediction for *KIRREL3* highlights a distinct regulatory logic compared with canonical IMF genes such as *LRP2BP* or *GAA*. Rather than directly controlling lipid synthesis, KIRREL3 appears to influence marbling visibility through a structural “adhesion barrier” mechanism: high expression seals myoblast membranes and restricts preadipocyte infiltration, producing high chemical IMF but poor visible marbling (low BMS); conversely, reduced expression (or virtual KO) loosens this barrier, permitting adipocyte redistribution and improving meat grade despite lower absolute IMF. This expression–phenotype decoupling suggests that *KIRREL3* may serve as a unique target for marker-assisted selection in Woking where the goal is not merely to maximize IMF content but to optimize its spatial patterning. Nevertheless, these predictions remain computational and require validation through CRISPR-Cas9 knockout in bovine myoblast–preadipocyte co-culture systems or allele-specific expression analysis in independent Woking populations.

### 4.6. Limitations and Future Perspectives

The main association cohorts provided sufficient power (*n* = 226 at 36 months and *n* = 204 at 48 months) for detecting moderate-to-large genetic effects. However, the independent validation group (*n* = 41) was relatively small. It may be underpowered for complex quantitative traits such as IMF content and fatty acid composition. Power calculations suggest limited statistical power. A sample of this size achieves approximately 0.55–0.60 power to detect a moderate effect (Cohen’s d ≈ 0.5) at α = 0.05. This falls below the conventional 0.80 threshold. These 41 animals were recruited from an independent batch. They came from the same commercial farm (Haoyue Cattle Farm, Changchun, China). They were entirely distinct from the main association populations. This design reduces within-study selection bias. It also improves external validity. Nevertheless, the validation results should be interpreted cautiously. Positive findings offer preliminary support for the robustness of the identified markers. Non-significant trends do not necessarily refute the associations observed in the larger cohorts.

Second, the causal regulatory mechanism of the identified SNPs remains unconfirmed at the molecular level. The intronic variants may exert direct cis-regulatory effects, or they may simply be in linkage disequilibrium with the true functional mutation(s). Luciferase reporter gene assays and EMSA experiments were not performed in the present study. Therefore, whether the variants alter transcription-factor binding or enhancer activity remains unknown. Consequently, the associations reported here are statistical, not causal, and should be interpreted accordingly.

Third, RT-qPCR relied on *β-actin* as a single reference gene; inter-tissue comparisons would benefit from geNorm-validated reference panels [43].

Fourth, the association tests reported nominal *p* values from pre-specified pairwise contrasts without formal family-wise error correction (e.g., Bonferroni or FDR). This approach is common in candidate-gene studies testing focused biological hypotheses. However, the reported associations should be interpreted as exploratory. They require confirmation in larger, independently analyzed cohorts.

Future work should prioritize three directions: (1) Functional validation. Dual-luciferase reporter assays should compare the 63 bp C and T alleles in bovine myoblast and preadipocyte lines. These assays are needed to confirm differential transcriptional activity. EMSA should also be performed. It can test whether the intronic variant alters transcription-factor binding. (2) Cellular niche identification. In situ hybridization or single-cell RNA sequencing of bovine longissimus dorsi should resolve whether *KIRREL3* is expressed by muscle fibers, intramuscular adipocytes, or innervating nerve terminals. This would directly test our hypothesis that adhesion-barrier modulation drives marbling pattern. (3) Independent large-scale validation. The 63 bp marker should be genotyped in an expanded Woking cohort (*n* > 500). Full feed-intake records should be collected. These data will estimate marker-assisted selection response. They will also test for epistatic interactions with *SCD*, *FASN*, and *PPARγ* loci. Environmental factors such as exact dietary composition and seasonal variation should be recorded as covariates. Future studies will also aim to expand the validation cohort, adding approximately 100–200 additional heads from subsequent batches of the same farm. This would substantially increase statistical power. Finally, vitamin A metabolism links to IMF deposition via FABP4 [44]. Exploring whether KIRREL3 expression responds to retinoid signaling may reveal a nutritional intervention point. Such an intervention could optimize expression of the favorable T allele. Song et al. [44] reported that vitamin A mediates *FABP4* to regulate IMF deposition. This finding provides a new target for optimizing beef quality. Fink et al. [45] functionally confirmed *PLAG1* as a pleiotropic candidate causal gene. It affects both body weight and milk characteristics. Their validation strategy provides a methodological reference for subsequent functional studies.

## 5. Conclusions

Two intronic *KIRREL3* SNPs (g.29756010C>T and g.29756149C>T) were identified in Woking cattle. Both loci were moderately polymorphic and in Hardy–Weinberg equilibrium. The 63-bp site associated with dressing percentage, rib thickness, and meat grade at both 36 and 48 months. The genetic pattern shifted over time: TT showed superiority at 36 months, while by 48 months, T-dominance emerged (TC ≈ TT > CC). The 203-bp site showed similar associations at 36 months that weakened by 48 months. In the validation cohort, CC animals accumulated more intramuscular fat and exhibited higher muscle *KIRREL3* expression than TT, yet paradoxically received lower meat grades. Five fatty acids differed significantly among genotypes after Benjamini–Hochberg correction (q < 0.05), including cis-10-pentadecenoic acid (C15:1), γ-linolenic acid (C18:3n6), cis-11,14,17-eicosatrienoic acid (C20:3n3), erucic acid (C22:1), and nervonic acid (C24:1); the dominant fatty acids (C16:0, C18:1n9c, C18:0, C18:2n6c) showed no significant differences. *KIRREL3* was most highly expressed in the kidney; muscle and adipose tissue showed intermediate levels.

These findings support the 63-bp SNP as a practical candidate marker for meat quality in local Northeast Chinese cattle, given its cross-stage persistence and its presence in Prairie Red and Yanbian cattle. However, this study establishes statistical association, not causation. The intronic variants may tag linked regulatory loci rather than exert direct effects, which means they may not directly alter protein function. The validation sample was small (*n* = 41), and the cohorts were raised under commercial conditions across different years. Confirmation in larger multi-farm populations is essential before integrating this marker into marker-assisted selection programs, together with functional assays such as luciferase reporter assays, EMSA, and single-cell resolution of *KIRREL3* expression in muscle.

## Figures and Tables

**Table 1:** Composition and nutrient levels of the basal diet (air-dry basis, %).

Ingredient	Content	Nutrient Level	Content
Corn	54.9	Moisture	14.00
Oats	20.00	Crude protein	12.00
Soybean meal	9.00	Lysine	0.35
Wheat bran	8.00	Crude fiber	12.00
Heat-treated soybean	3.00	Calcium	0.40–2.20
Alfalfa pellets	2.50	Phosphorus	0.35
Salt	0.50	Crude ash	15.00
Dicalcium phosphate	0.10	Sodium	0.50–3.00
Premix	2.00		
Total	100.00		

Note: The premix provided per kilogram of diet: VA 3,000 IU, VD_3_ 500 IU, VE 20 IU, Fe 50 mg, Cu 10 mg, Mn 40 mg, Zn 40 mg, I 0.5 mg, Se 0.3 mg, Co 0.1 mg (adjustable according to actual formulation). Values represent approximate proportions due to commercial confidentiality.

**Table 2:** Forward and reverse primers for real-time quantitative PCR for specific gene mRNA.

Gene	Application	Primer Sequence (5′ → 3′)	Product Length (bp)	Annealing Temperature (°C)
*KIRREL3*	PCR amplification	F: GCAGCCTTTTTCCAACACGT	603	56
		R: ATCAGCTGGCACCTTGGATC		
*KIRREL3*	RT-qPCR	F: CCTCAACCTCACCTGCCATG	102	60
		R: AGGGGTCTTGGAGTAGGTGGC		
*β-actin*	RT-qPCR (reference)	F: GTCCACCTTCCAGCAGAT	96	60
		R: GCTAACAGTCCGCCTAGAA		

**Table 3:** SNP locus polymorphism and genetic parameters.

SNP Site	Genotype	*n*	Frequency	Allele	Frequency	Ho	He	Ne	PIC	χ^2^	*p*-Value
**g.29756010C>T (63-bp site)**	CC	66	0.29	C	0.55	0.52	0.49	1.98	0.37	0.48	>0.05
	TC	117	0.52	T	0.45						
	TT	43	0.19								
**g.29756149C>T (203-bp site)**	CC	65	0.32	C	0.58	0.53	0.49	1.95	0.37	1.62	>0.05
	TC	108	0.53	T	0.42						
	TT	31	0.15								

Note: The 63-bp site was genotyped in the 36-month cohort (*n* = 226). The 203-bp site was genotyped in the 48-month cohort (*n* = 206); 204 samples yielded successful calls (call rate = 99.0%). Ho, observed heterozygosity; He, expected heterozygosity; Ne, effective number of alleles; PIC, polymorphism information content.

**Table 4:** Association between SNP locus genotypes and traits of 36-month-old beef cattle.

	*KIRREL3* g.29756010C>T				*KIRREL3* g.29756149C>T			
	CC	TC	TT	*p*-Value	CC	TC	TT	*p*-Value
Live weight before slaughter (kg)	791.79 ± 91.09	800.05 ± 92.42	797.26 ± 85.57	>0.05	786.20 ± 89.07	799.73 ± 92.62	805.66 ± 87.58	>0.05
Carcass weight (kg)	479.46 ± 69.38	484.36 ± 60.41	484.94 ± 60.26	>0.05	477.33 ± 70.82	484.84 ± 60.83	491.26 ± 69.59	>0.05
Dressing percentage (%)	59.98 ± 1.71 ^b^	60.50 ± 1.86 ^ab^	61.01 ± 1.90 ^a^	<0.05	60.13 ± 1.85 ^b^	60.58 ± 1.92 ^ab^	61.31 ± 1.82 ^a^	<0.05
Subcutaneous fat thickness (cm)	2.87 ± 0.66	2.88 ± 0.66	3.11 ± 0.72	>0.05	2.91 ± 0.63	2.90 ± 0.68	3.12 ± 0.62	>0.05
Loin eye area (cm^2^)	39.29 ± 4.63	40.42 ± 6.65	38.86 ± 6.35	>0.05	39.33 ± 5.05	40.77 ± 6.62	38.81 ± 5.02	>0.05
Grade	2.32 ± 0.90 ^b^	2.23 ± 1.00 ^b^	2.84 ± 0.87 ^a^	<0.05	2.29 ± 0.90 ^b^	2.23 ± 1.00 ^b^	2.72 ± 0.91 ^a^	<0.05
Rib meat thickness (cm)	6.90 ± 0.69 ^b^	7.18 ± 1.04 ^b^	7.48 ± 0.98 ^a^	<0.05	6.91 ± 0.76 ^b^	7.25 ± 1.07 ^b^	7.56 ± 0.74 ^a^	<0.05
Intermuscular fat thickness (cm)	4.60 ± 0.68 ^b^	4.65 ± 0.79 ^ab^	4.93 ± 0.81 ^a^	<0.05	4.65 ± 0.70	4.69 ± 0.81	4.65 ± 0.69	>0.05

Values are presented as mean ± standard deviation. Within each row, means with different superscript letters differ significantly (*p* < 0.05); the letter “a” denotes the highest mean, followed by “b”, etc. Means sharing a letter (e.g., “a” and “ab”, or “b” and “ab”) do not differ significantly. Means without superscript letters were not significantly different (*p* > 0.05).

**Table 5:** Association between SNP locus genotypes and traits of 48-month-old beef cattle.

	*KIRREL3* g.29756010C>T				*KIRREL3* g.29756149C>T			
	CC	TC	TT	*p*-Value	CC	TC	TT	*p*-Value
Live weight before slaughter (kg)	823.59 ± 68.27	820.10 ± 68.68	811.78 ± 70.98	>0.05	812.05 ± 59.48	820.83 ± 72.30	816.96 ± 61.94	>0.05
Carcass weight (kg)	498.68 ± 45.26	504.84 ± 46.13	500.39 ± 46.85	>0.05	492.92 ± 44.10	504.97 ± 46.84	504.10 ± 43.04	>0.05
Dressing percentage (%)	60.53 ± 2.07 ^b^	61.34 ± 2.18 ^ab^	61.63 ± 1.72 ^a^	<0.05	60.64 ± 1.65 ^b^	61.32 ± 2.20 ^ab^	61.68 ± 1.77 ^a^	<0.05
Subcutaneous fat thickness (cm)	2.83 ± 0.72 ^ab^	3.20 ± 0.76 ^a^	2.89 ± 0.80 ^b^	<0.05	2.98 ± 0.68	3.14 ± 0.76	2.97 ± 0.82	>0.05
Loin eye area (cm^2^)	41.85 ± 5.95	41.17 ± 5.86	41.43 ± 6.82	>0.05	40.29 ± 5.45	41.22 ± 6.11	41.97 ± 6.37	>0.05
Grade	2.12 ± 0.60 ^b^	2.75 ± 0.94 ^a^	2.80 ± 1.09 ^a^	<0.05	2.47 ± 0.62 ^b^	2.72 ± 0.97 ^b^	2.88 ± 1.05 ^a^	<0.05
Rib meat thickness (cm)	7.16 ± 0.85 ^b^	7.69 ± 0.73 ^a^	7.75 ± 0.66 ^a^	<0.05	7.38 ± 0.68 ^b^	7.65 ± 0.77 ^ab^	7.81 ± 0.66 ^a^	<0.05
Intermuscular fat thickness (cm)	4.86 ± 0.66	5.01 ± 0.73	4.93 ± 0.75	>0.05	4.91 ± 0.59	4.99 ± 0.75	4.97 ± 0.72	>0.05

Values are presented as mean ± standard deviation. Within each row, means with different superscript letters differ significantly (*p* < 0.05, Tukey’s HSD); the letter “a” denotes the highest mean, followed by “b”, etc. Means sharing any letter do not differ significantly. Means without superscript letters were not significantly different (*p* > 0.05).

**Table 6:** Fatty acid composition (%) of *KIRREL3*.

	CC (*n* = 12)	TC (*n* = 20)	TT (*n* = 9)	q-Value
Lauric acid (C12:0)	0.0887 ± 0.0213	0.1037 ± 0.0387	0.0876 ± 0.0280	0.9876
Tridecanoic acid (C13:0)	ND	0.0025 ± 0.0049	0.0007 ± 0.0022	–
Myristic acid (C14:0)	4.7643 ± 0.5799	4.9095 ± 0.4425	4.9977 ± 0.2755	0.8987
Pentadecanoic acid (C15:0)	1.6104 ± 0.1399	1.4928 ± 0.2385	1.4058 ± 0.1905	0.293
Cis-10-pentadecenoic acid (C15:1)	0.3668 ± 0.0302 ^a^	0.2937 ± 0.0278 ^b^	0.2635 ± 0.0720 ^b^	0.0194
Palmitic acid (C16:0)	18.3560 ± 1.1656	16.7570 ± 0.8333	17.8633 ± 1.2474	0.4401
Palmitoleic acid (C16:1)	10.5740 ± 0.6532	10.6379 ± 1.1515	9.8959 ± 1.2347	0.6235
Heptadecanoic acid (C17:0)	3.9567 ± 0.0787	3.4148 ± 0.4605	3.0312 ± 0.4038	0.1143
Cis-10-heptadecenoic acid (C17:1)	2.4511 ± 0.2085	2.2898 ± 0.2716	1.9359 ± 0.3200	0.3051
Stearic acid (C18:0)	15.6009 ± 0.4083	15.7211 ± 1.0559	15.7276 ± 1.7950	0.8782
Elaidic acid (C18:1n9t)	1.6939 ± 0.1429	1.8708 ± 0.2703	1.8571 ± 0.1988	0.5108
Oleic acid (C18:1n9c)	22.5268 ± 2.5455	24.2109 ± 1.5368	25.3261 ± 3.3393	0.9959
Linoelaidic acid (C18:2n6t)	0.5201 ± 0.3806	0.6478 ± 0.3631	0.3656 ± 0.1812	0.2257
Linoleic acid (C18:2n6c)	11.2106 ± 0.3611	11.0657 ± 1.0011	10.4983 ± 1.2473	0.4083
γ-Linolenic acid (C18:3n6)	0.0892 ± 0.0250 ^b^	0.1264 ± 0.0300 ^a^	0.1171 ± 0.0322 ^ab^	0.0282
α-Linolenic acid (C18:3n3)	1.0715 ± 0.0503	0.9488 ± 0.1277	0.9394 ± 0.1599	0.3075
Arachidic acid (C20:0)	0.1368 ± 0.0239	0.1301 ± 0.0156	0.1339 ± 0.0377	0.7794
Cis-11-eicosenoic acid (C20:1)	0.1103 ± 0.0206	0.0961 ± 0.0229	0.1065 ± 0.0380	0.4565
Cis-11,14-eicosadienoic acid (C20:2)	0.1707 ± 0.0636	0.1633 ± 0.0523	0.1692 ± 0.0765	0.4653
Heneicosanoic acid (C21:0)	0.0488 ± 0.0111	0.0411 ± 0.0075	0.0410 ± 0.0114	0.5369
Dihomo-γ-linolenic acid (C20:3n6)	0.2716 ± 0.0630	0.3041 ± 0.0566	0.3249 ± 0.0936	0.9792
Arachidonic acid (C20:4n6)	0.0444 ± 0.0075	0.0362 ± 0.0124	0.0447 ± 0.0167	0.0667
Cis-11,14,17-eicosatrienoic acid (C20:3n3)	0.0733 ± 0.0479 ^a^	0.0364 ± 0.0090 ^ab^	0.0445 ± 0.0147 ^b^	0.0008
Eicosapentaenoic acid (C20:5n3)	0.0264 ± 0.0047	0.0230 ± 0.0024	0.0263 ± 0.0054	0.2502
Behenic acid (C22:0)	0.0063 ± 0.0039	0.0061 ± 0.0028	0.0047 ± 0.0036	0.3977
Erucic acid (C22:1)	0.3640 ± 0.2343 ^b^	0.5353 ± 0.2489 ^a^	0.6740 ± 0.1127 ^a^	0.0067
Cis-13,16-docosadienoic acid (C22:2)	0.7669 ± 0.0821	0.7628 ± 0.1649	0.8476 ± 0.2600	0.5218
Tricosanoic acid (C23:0)	0.0170 ± 0.0039	0.0179 ± 0.0030	0.0165 ± 0.0047	0.4109
Lignoceric acid (C24:0)	0.0072 ± 0.0031	0.0064 ± 0.0021	0.0053 ± 0.0023	0.2192
Docosahexaenoic acid (C22:6n3)	0.0513 ± 0.0024	0.0613 ± 0.0110	0.0566 ± 0.0102	0.7343
Nervonic acid (C24:1)	0.0402 ± 0.0107 ^a^	0.0220 ± 0.0076 ^b^	0.0273 ± 0.0094 ^b^	0.0000
Decanoic acid (C10:0)	0.0401 ± 0.0180	0.0626 ± 0.0259	0.0530 ± 0.0194	0.1167

ND: Not detected. Values are expressed as relative weight percentage (% of total identified fatty acids) based on fresh tissue weight. Statistical analyses were performed on raw concentrations (g/100g). Within each row, means with different superscript letters differ significantly (q < 0.05, Dunn’s test with Benjamini–Hochberg correction); the letter “a” denotes the highest mean, followed by “b”, etc. Means sharing any letter (e.g., “a” and “ab”, or “b” and “ab”) do not differ significantly. Means without superscript letters were not significantly different (q > 0.05).

**Table 7:** Meat quality detection results of different genotypes of KIRREL3 gene.

Traits	CC (*n* = 12)	TC (*n* = 20)	TT (*n* = 9)	*p*-Value
Pressurized water loss rate/%	27.79 ± 3.26	29.23 ± 2.32	30.71 ± 3.13	0.0717
Cooked meat rate/%	66.09 ± 6.83	64.05 ± 2.74	62.93 ± 3.18	0.2467
Meat tenderness/N	23.70 ± 7.81	29.68 ± 10.51	30.43 ± 8.30	0.0784
Centrifugal loss/%	23.20 ± 2.19 ^b^	24.39 ± 2.56 ^ab^	25.89 ± 1.92 ^a^	0.0440
Meat color L value	42.58 ± 4.93 ^a^	36.97 ± 5.04 ^b^	34.99 ± 2.55 ^b^	0.0010
Meat color a value	18.48 ± 1.48	17.68 ± 3.23	16.61 ± 2.40	0.2936
Meat color b value	8.49 ± 2.20 ^a^	6.86 ± 2.19 ^ab^	5.26 ± 1.20 ^b^	0.0074
pH value	5.43 ± 0.23	5.36 ± 0.24	5.31 ± 0.18	0.3740
Drip loss 24 h/%	0.49 ± 0.20	0.56 ± 0.18	0.67 ± 0.12	0.0700
Intramuscular fat content/%	21.70 ± 6.24 ^a^	17.50 ± 5.66 ^ab^	15.29 ± 3.05 ^b^	0.0267
Moisture content/%	53.67 ± 7.67	58.20 ± 6.23	60.00 ± 4.30	0.0826
Cooking loss/%	13.51 ± 2.87 ^b^	16.90 ± 2.55 ^a^	16.45 ± 3.49 ^ab^	0.0078
Protein/%	19.66 ± 1.10 ^b^	20.70 ± 1.32 ^ab^	21.59 ± 0.60 ^a^	0.0018

Values are presented as mean ± standard deviation. Within each row, means with different superscript letters differ significantly (*p* < 0.05, Tukey’s HSD); the letter ‘a’ denotes the highest mean, followed by ‘b’, etc. Means sharing any letter (e.g., a and ab, or b and ab) do not differ significantly. Means without superscript letters were not significantly different (*p* > 0.05).

**Table 8:** Genetic diversity parameters of *KIRREL3* gene SNP loci in Prairie Red cattle and Yanbian cattle populations.

Project	Genotype Frequency			Allele Frequency		Ho	He	Ne	PIC	χ^2^	*p*-Value
	Wild Type (CC)	Heterozygous Type (TC)	Mutant Type (TT)	Wild Type (C)	Mutant Type (T)						
Prairie Red cattle	0.16 (8)	0.50 (25)	0.34 (17)	0.41	0.59	0.50	0.48	1.94	0.37	0.06	>0.05
Yanbian cattle	0.26 (13)	0.52 (26)	0.22 (11)	0.52	0.48	0.52	0.50	2.00	0.37	0.09	>0.05

Note: The numbers in parentheses indicate the number of individuals; Ho: Observed heterozygosity; He: Expected heterozygosity; Ne: Effective number of alleles; PIC: Polymorphism information content; χ^2^: Chi-square value of the Hardy–Weinberg equilibrium test. Both sites conform to Hardy–Weinberg equilibrium (*p* > 0.05) and belong to moderately polymorphic sites (0.25 < PIC < 0.50).

## Data Availability

The raw sequencing chromatograms and phenotypic datasets generated during this study are available from the corresponding author upon reasonable request. The data are not publicly available due to commercial breeding confidentiality agreements. Accession numbers for deposited sequence data will be provided upon acceptance.

## Abbreviations

The following abbreviations are used in this manuscript:

IMF	Intramuscular fat
MAS	Marker-assisted selection
GS	Genomic selection
PIC	Polymorphism information content
He	Expected heterozygosity
Ho	Observed heterozygosity
Ne	Effective allele number
HWE	Hardy–Weinberg equilibrium
SFA	Saturated fatty acid
MUFA	Monounsaturated fatty acid
PUFA	Polyunsaturated fatty acid
UFA	Unsaturated fatty acid
RT-qPCR	Real-time quantitative PCR
